# Exploiting the Complexities of Glioblastoma Stem Cells: Insights for Cancer Initiation and Therapeutic Targeting

**DOI:** 10.3390/ijms21155278

**Published:** 2020-07-25

**Authors:** Joana Vieira de Castro, Céline S. Gonçalves, Adília Hormigo, Bruno M. Costa

**Affiliations:** 1 Life and Health Sciences Research Institute (ICVS), School of Medicine, University of Minho, Campus Gualtar, 4710-057 Braga, Portugal; joana.vieira.castro@gmail.com (J.V.d.C.); celinegoncalves@med.uminho.pt (C.S.G.); 2 ICVS/3B’s—PT Government Associate Laboratory, 4710-057 Braga/Guimarães, Portugal; 3 Department of Neurology, Neurosurgery, Medicine, The Tisch Cancer Institute and Icahn School of Medicine at Mount Sinai, NY 10029-6574, USA; adilia.hormigo@mssm.edu

**Keywords:** cancer heterogeneity, GSCs microenvironment, molecular pathways, stem cell markers, therapy resistance

## Abstract

The discovery of glioblastoma stem cells (GSCs) in the 2000s revolutionized the cancer research field, raising new questions regarding the putative cell(s) of origin of this tumor type, and partly explaining the highly heterogeneous nature of glioblastoma (GBM). Increasing evidence has suggested that GSCs play critical roles in tumor initiation, progression, and resistance to conventional therapies. The remarkable oncogenic features of GSCs have generated significant interest in better defining and characterizing these cells and determining novel pathways driving GBM that could constitute attractive key therapeutic targets. While exciting breakthroughs have been achieved in the field, the characterization of GSCs is a challenge and the cell of origin of GBM remains controversial. For example, the use of several cell-surface molecular markers to identify and isolate GSCs has been a challenge. It is now widely accepted that none of these markers is, per se, sufficiently robust to distinguish GSCs from normal stem cells. Finding new strategies that are able to more efficiently and specifically target these niches could also prove invaluable against this devastating and therapy-insensitive tumor. In this review paper, we summarize the most relevant findings and discuss emerging concepts and open questions in the field of GSCs, some of which are, to some extent, pertinent to other cancer stem cells.

## 1. Introduction: Cancer Heterogeneity and Tumor-Initiation Models

For decades, the concept of tumor heterogeneity related mostly to the presence of transformed and normal host cells within a tumor lesion. Differences between tumors were mostly attributed to infiltration of tumor cells into the surrounding tissue or vice versa, and were considered to be the result of stochastic events [1]. Over the years, more precise methodological and technological approaches have revealed that intrinsic tumor heterogeneity is one of the key features of tumorigenesis, largely responsible for tumor progression, resistance to therapy, and relapse. Tumor heterogeneity gives an evolutionary advantage in overcoming the selective pressures imposed by microenvironmental oscillations and/or exposure to therapies, most commonly chemotherapy and radiotherapy. Indeed, the presence of pre-existent tumor clones intrinsically insensitive to therapies is one of the main causes of treatment failure and subsequent tumor recurrence. Therefore, understanding the mechanisms underlying tumor heterogeneity is an essential step in developing better precision therapies, with significant potential benefits particularly in the case of notoriously therapy-refractory cancers.

To date, two main models explaining the origin of cancer cells’ heterogeneity have been proposed [2]. In 1976, Nowell and coworkers presented the clonal evolution model, postulating that cancer is generated through an evolutionary process, wherein tumors accumulate (epi)genetically and phenotypically diverse cell subpopulations. According to this model, (epi)genetic mutations can generate randomly, and any new phenotypes are subjected to pressures of natural selection, under which the most adapted cells are able to survive and proliferate [3]. This variability would be critical when cancer cells encounter environmental changes, such as those induced by chemotherapy or radiotherapy, in which the acquisition of a resistant clone/phenotype would allow a subpopulation of cells to survive, expand, and dominantly repopulate the tumor [3]. More recently, the cancer stem cell (CSC) model has become a widely accepted theory of cancer initiation, progression, and resistance to therapy, emphasizing the importance of various levels of differentiation in cancer cells, in which the most undifferentiated ones are capable of generating other phenotypically distinct cells in a unidirectional manner [4,5]. This theory postulates a hierarchical organization in which a tumor generates from a minority of cells with stem-cell characteristics, known as CSCs. By asymmetric division, these cells can maintain their population by self-renewal, and generate more differentiated cells with limited proliferation capacity that constitute the majority of the tumor bulk. In this view, heterogeneity is seen as the dualistic nature of CSCs (tumorigenic) and non-CSCs with various degrees of differentiation (non-tumorigenic), regardless of their genetic background [6], presumably determined by epigenetic changes [7]. Although the clonal evolution and the CSC models may seem to be mutually exclusive, they can indeed coexist and both premises can explain the origin of tumor heterogeneity. For example, following the CSC model (i.e., hierarchically organized into epigenetically distinct populations of cancer cells—tumorigenic and non-tumorigenic cells), CSCs in tumors are also expected to undergo clonal evolution during tumor progression (reviewed by Kreso and Dick [8]). Indeed, genetically diverse CSCs populations have been observed in some tumors [9,10,11,12,13].

Cell plasticity in response to microenvironmental cues such as blood vessel density, differences in oxygen pressures, and compositions of the extracellular matrix can generate tumor heterogeneity. Therefore, the CSC model can be updated with the concept of various degrees of “stemness” and/or tumorigenic potential, determined both by stochastic events and microenvironmental cues [14,15].

Even though the CSC theory has been widely accepted, the origin of CSCs remains a mystery. Indeed, while the term “stem cell” has been appropriated from the normal stem/progenitor cells, they are not necessarily at the origin of the CSCs. Two distinct hypotheses for their origin are being considered: (i) a normal stem cell or progenitor cell that undergoes specific genetic aberrations; or (ii) de-differentiation of differentiated cells into cells with stem-cell phenotypes. Stem cells produce transient cells, which in turn generate lineage-restricted progeny that become differentiated effector cells. In fact, normal stem cells or progenitor cells could be ideal candidates for malignant transformation since they represent the most primitive cells, live longer, and typically re-enter cell division to replace the pool of both stem cells and differentiated progenies. Therefore, in theory, these stem/progenitor cells could accumulate sequential genetic or epigenetic mutations that eventually lead to oncogenesis.

Overall, it has been widely postulated that the eradication of CSCs is necessary to interrupt tumor expansion or prevent regrowth after therapy [16]. A better understanding of the molecular and functional characteristics of the subpopulation of cancer stem cells will potentially allow the development of more effective therapies for various malignant tumors. This is indeed an urgent unmet need for therapies for brain tumors, particularly glioblastoma (GBM), for which no effective therapies are available. Identifying better methods of detecting glioblastoma CSCs (GSCs), and refining their isolation and culture, is a first critical step in this effort. In this review, we summarize the most widely accepted biomarkers for GSC identification, and discuss the major signaling pathways that have been associated with GSC maintenance and may represent novel potential therapeutic targets.

## 2. Glioblastoma (GBM) and Putative Cells of Origin

GBM (WHO Grade IV glioma) is the most common primary brain tumor in adults, with a very dismal prognosis (median survival of approximately 15 months) [17]. It is a highly heterogeneous tumor at the cellular and molecular levels, a consequence of genetic and/or epigenetic causes and environmental factors [18,19]. Several studies have attempted to identify the most relevant cell of origin of GBMs, testing whether the two hypotheses described for other cancer types may also be applicable in these brain tumors. According to studies based on transgenic animal models, the hypothesis is that neural stem/progenitor cells (NSPCs) in the brain are the primary cellular targets for gliomagenesis [16], utilizing NSPC-related cell promoters such as NESTIN and glial fibrillary acidic protein (GFAP) to inactivate tumor suppressors (e.g., *PTEN* or *TP53*) or drive oncogene expression (e.g., activated *RAS*) in specific cellular niches. These models are effective in initiating cellular transformation and driving oncogenesis [20,21,22,23,24]. Moreover, differentiated cells in the central nervous system (CNS; neurons and astrocytes) can drive tumorigenesis upon oncogenic transformation [25]. Additionally, several studies have demonstrated that oligodendrocyte precursor cells (OPCs) can also be cells of origin for malignant gliomas, as they are susceptible to transformation by a wide range of mutations often found in human gliomas, including mutant forms of PTEN, NF1, RAS, and TP53 [26,27]. Globally, these studies suggest that various cells in the brain can serve as cells of origin for CNS tumors, and emphasize a capacity for interconversion between GSCs and differentiated cancer cells during tumor initiation and maintenance [26,28]. Therefore, it is critical to elucidate the molecular mechanisms behind this plastic behavior to develop more effective therapies for GBM, as well as to explore how current chemotherapies and radiotherapies can potentially influence this process. Whether a particular cell type and/or differentiation state is more frequently targeted for oncogenic transformation in different subtypes of glioma also remains an open question.

## 3. Glioblastoma Stem Cells (GSCs)

In 2000, Uchida and coworkers isolated human NSPCs using PROM1 (widely known as CD133) [29]. Prominin-1 is a 120 kDa five-transmembrane cell-surface protein of unknown function expressed by neural stem cells (NSCs), adult ependymal cells, and endothelial precursor cells [30]. Brain-tumor stem cells (BTSCs) were initially isolated from primary tumors by cell sorting based on CD133 expression [31]. Functionally, these CD133^+^ tumor cells generated neurospheres, had self-renewal capacity and a high proliferation potential, and were multipotent [31]. Additionally, CD133^+^ BTSCs displayed a remarkable in vivo tumorigenicity when implanted into immunodeficient mice [32]. Indeed, as few as 100 CD133^+^ tumor cells were able to originate tumors that recapitulated the parental tumor, whereas 100,000 CD133^−^ cells did not have that capacity [32]. These results provided strong evidence for a key role of CD133^+^ GSCs in brain tumor biology. Several subsequent studies implicated them in resistance to radiotherapy and chemotherapy [33,34]. The capacity of these tumors to recur after treatment was linked to specific characteristics of GSCs, such as quiescent phenotype, enhanced DNA repair capacity, preferential activation of DNA damage checkpoint responses [33], and increased expression of drug efflux pumps and antiapoptotic proteins [35]. Nonetheless, the expression of CD133 on the cell surface does not seem to be a requirement for neurosphere formation. Indeed, CD133^−^ cells isolated from glioma specimens can have stem-cell-like characteristics, although with lower efficiency, and similar tumorigenic potential [36,37]. Moreover, it has been shown that CD133^+^ cells may lack GSC-like features, while other cell types, including normal endothelial cells and endothelial glioma cells, express CD133 [30]. These findings suggest that the subpopulation of GSCs within heterogeneous cell populations of GBM must be specifically targeted in combination with currently available therapies in order to achieve a more efficient and long-lasting clinical response.

It is now accepted that GSCs also present remarkable heterogeneity, as reflected by the presence of diverse GBM clones [38]. This can be influenced by their location within the tumor and the multiple microenvironmental clues originating from different surrounding cells, which also change during the various stages of tumor initiation, progression, and recurrence [39,40,41]. GSCs show a mixture of cellular morphologies when cultured as neurospheres [42,43], and CD133^+^ and CD133^−^ GSCs have been shown to be able to convert into each other within one GBM [38,44]. It is noteworthy that the intertumoral heterogeneity of GSCs could also be taken into account in the molecular classification of GBM if it becomes clear that different GBMs present enrichment of particular GSC subtypes. Indeed, some studies have proposed similarities of the transcriptional profile of CD133^−^ GSCs with the mesenchymal subtype and adult NSPCs, and CD133^+^ GSCs with the proneural subtype and fetal NSPCs [37,45]. In line with this, it was described that GSC lines grown as adherent monolayers were transcriptionally more similar to fetal NSPCs than to adult ones [46]. Moreover, studies by Verhaak, et al. [47] raised the hypothesis of the existence of (i) a potential common cell of origin for all GBM subtypes (proneural, neural, mesenchymal, and classical), which at some point follow distinct differentiation paths; or (ii) the existence of different cells of origin for each subtype. Indeed, for example, the classical subtype frequently presents the expression of the neural precursor and stem-cell marker *NES*, while the proneural subtype is also associated with progenitor or neural stem cells with an enrichment in oligodendrocytic and proneural development genes (e.g., *PDGFRA*, *OLIG2*, *SOX*, and *TCF4*) [47]. This suggests that the heterogeneity of GBM reflects the heterogeneity of GSCs. Suvà, et al. [48] identified a set of four transcription factors (POU3F2, SOX2, SALL2, and OLIG2) in the proneural subtype that are able to reprogram differentiated tumor cells into GSCs. These transcription factors are required to maintain the tumor-forming capacity of these cells, suggesting that mediators of stem-cell programs could drive the oncogenic capacity of GSCs [48]. Interestingly, using single-cell RNA sequencing, Patel, et al. [49] identified novel genes predominately present in GSCs as compared to their differentiated counterparts from the same GBM tumor [49]. Moreover, stemness gradient and cell-cycle signatures have an inverse correlation, suggesting that stem cells divide more slowly than differentiated tumor cells [49]. Another recent study, using single-cell functional analysis of GBM patient samples, showed that individual clones have unique proliferation and differentiation abilities, as well as a remarkable genomic variation and different responses to therapy [50], suggesting that functional clonal profiling could be used to identify drug-resistant tumor clones, potentially leading to the discovery of novel treatments. In contrast with differentiated GBM cells, which metabolically prefer aerobic glycolysis (mainly known as the Warburg effect), GSCs are considered highly flexible and can switch from aerobic glycolysis to oxidative phosphorylation as an adaptation mechanism (as reviewed by Garnier, et al. [51]). In fact, additional studies have demonstrated that distinct GSC clones, even from the same tumor, could display variability in gene expression profile and metabolic dependencies [52,53], adding support to the concept of intratumor GSC heterogeneity.

### 3.1. Identification, Isolation, and Propagation of GSCs

**Figure 1:**
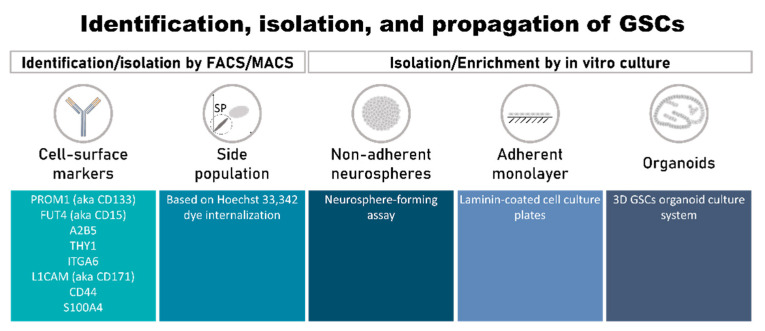
Methods used for glioma stem cell (GSC) identification, isolation, and propagation. GSCs can be identified and isolated either by fluorescence-activated cell sorting (FACS) or magnetic-activated cell sorting (MACS) based on their expression of cell-surface markers (e.g., CD133, CD15, CD90, and A2B5), or based on the differential efflux of Hoechst 33,342 dye by a multidrug-like transporter using the side-population (SP) assay. Additionally, GSCs can be enriched in vitro using three techniques: the neurosphere-forming assay, the culture of adherent monolayers in laminin-coated plates, or using 3D organoid culture systems.

Efforts have been made to discover, validate, and use GSC enrichment methods. However, the intrinsic heterogeneity of tumor specimens, the rarity of the GSC population, and the expression of cell-surface epitopes common to non-GSCs and other normal cell types have been major hurdles to specific isolation and propagation of GSCs [30]. To date, these cells have been mostly isolated by cell sorting, followed by in vitro enrichment using serum-free culture conditions supplemented with specific growth factors that allow for neurosphere formation (Figure 1).

#### 3.1.1. Cell-Surface Markers of GSCs

Most GSC markers have been appropriated from normal NSPCs, such as BMI1 [54], MSI1/2 [54], NANOG [48,55], NESTIN [56], and SOX2 [54], among others [57,58]. However, the use of intracellular proteins for GSC enrichment by fluorescence-activated cell sorting (FACS) or magnetic-activated cell sorting (MACS) presents limitations, with purity at separation of 79.3–96.7% or 46.9%–79.8%, respectively [31,59]. The major findings regarding cell-surface markers that have been used to isolate GSCs, including CD133, CD15, A2B5, CD90, L1CAM, and the combination of CD44 and ID1, are summarized below.

##### CD133 (Official Symbol: PROM1)

The number of CD133^+^ cells quantified by flow cytometry from human glioma samples, glioma sphere cultures, and established glioma cell lines varies from very low/rare [37,60] to as high as 60% [37,59,61]. This variation may be explained by the recognition of inconstant glycosylated epitopes by the current available antibodies (AC133 or AC141) [62]. Interestingly, Kemper, et al. [63] demonstrated that while the detection of AC133 epitope decreased with CSC differentiation, no effect was observed regarding the expressions of CD133 mRNA or protein (at the surface or not), the promoter activity, or splice variant. Moreover, the authors described that although AC133 did not recognize a glycosylated epitope, only differences in CD133 glycosylation occurred upon CSC differentiation, which might suggest that (i) glycosylation might be “hiding” the AC133 epitope, possibly due to a difference in CD133 folding; and (ii) only the glycosylated surface protein CD133 is CSC-dependent [63]. In glioma, the first association between CD133 and patients’ adverse clinical outcomes was reported in 2008 [61]. Soon after, therapies targeting CD133 were postulated to represent a promising strategy for GBM treatment. Brescia and colleagues demonstrated that inhibition of CD133 expression by short hairpin RNA in GBM-derived neurospheres impaired their self-renewal and tumorigenic capacity [64]. Additionally, it was shown that treatment with carbon nanotubes conjugated with anti-CD133 monoclonal antibody followed by irradiation with near-infrared laser light can selectively target CD133^+^ GBM cells, and the photothermolysis caused by the nanotubes can kill the targeted cells [65]. More recently, Emlet, et al. [66] demonstrated that EGFRvIII is highly co-expressed with CD133, and that the EGFRvIII^+^/CD133^+^ population presented increased self-renewal and tumor-initiating ability. By using a bispecific antibody, they could eliminate the EGFRvIII^+^/CD133^+^ population, reducing the tumorigenicity of implanted tumor cells [66]. Moreover, it was demonstrated that the expression of CD133 could be regulated at the level of cell cycle, with potentially slow-cycling NSPCs lacking CD133 expression during G0/G1 cell cycle phase but still maintaining multipotency [67].

##### CD15 (Official Symbol: FUT4)

CD15 is a large carbohydrate antigen expressed at the surface of embryonic and adult NSPCs, in association with glycolipids and glycoproteins [59,68], thus representing a putative useful marker for GSCs. CD15 is also commonly termed SSEA-1 (stage-specific embryonic antigen-1) or LeX (Lewis-X Antigen). CD15^+^ cells are able of self-renewal and multilineage differentiation and have increased expression of the stem-cell markers BMI1 and SOX2 [59]. CD15^+^ cells isolated from GBMs are also highly tumorigenic, while CD15^−^ cells present limited tumor-formation capacity [69].

##### A2B5

The A2B5 monoclonal antibody recognizes ganglioside antigens that are expressed at the cell surface of NSCs isolated from the subventricular zone of human embryos [70], and by neural precursor cells from the subcortical white matter in the adult human brain [71]. In GBM and anaplastic astrocytoma, 33–90% of the cells express the A2B5 antigen [72]. Two different studies demonstrated that A2B5^+^ cells were able to form tumors in immunocompromised mice, while A2B5^−^ cells were not able to do so [70,72]. In addition, A2B5^+^/CD133^+^ and A2B5^+^/CD133^−^ subpopulations from glioma were capable of forming neurospheres in vitro and initiating tumors in vivo, suggesting that A2B5 is a GSC marker [70]. Another study showed that ST8SIA3, the enzyme that synthetizes the A2B5 glycolipid, increased A2B5 immunoreactivity, GBM cell proliferation, migration, and clonogenicity in vitro [73]. More importantly, ST8SIA3 silencing significantly increased the overall survival of a mouse GBM orthotopic model [73]. A cohort of genes and pathways significantly dysregulated in A2B5^+^ tumor progenitor cells, including *SIX1*, *EYA1*, and *DACH2*, were identified using A2B5, followed by messenger RNA profiling and comparison to A2B5^+^ from normal white matter [74]. This set of genes are mostly expressed during development and not during adult life, which makes them particularly attractive as selective therapeutic targets.

##### CD90 (Official Symbol: THY1)

Another potential marker for GSCs is CD90, an N-glycosylated glycophosphatidylinositol (GPI)-anchored cell-surface protein, a known marker for bone-marrow-derived and hematopoietic stem cells [75]. Recently, it was identified as a marker for human GSCs [76]. In GBM, 100% of the CD133^+^ cells co-express CD90, but only a small portion of CD90^+^ cells co-express CD133. Moreover, CD90 expression levels were significantly higher in high-grade than in low-grade gliomas [76].

##### ITGA6

Integrin-α6 (ITGA6) is a member of the integrin family of extracellular matrix receptors for laminin and platelets. In the brain, this receptor regulates GSC maintenance [77] and NSC growth [78]. In GBM biopsies, cells positive for integrin-α6 were localized close to the tumor vasculature and co-expressed the stem-cell markers CD133 and NESTIN [79]. FACS for integrin-α6 alone or in combination with CD133 led to an enrichment of cells with higher self-renewal capacity in vitro. Orthotopic injection of integrin-α6-positive cells into the brains of immunocompromised mice resulted in shorter survival when compared to integrin-α6-negative cells. Furthermore, shRNA-mediated knockdown of integrin-α6 or treatment with integrin-blocking antibody reduced both neurosphere formation in vitro and tumor growth in vivo [79]. These findings strongly indicate a role for integrin-α6 in GSCs’ self-renewal and maintenance.

##### CD171 (Official Symbol: L1CAM)

L1CAM is a neural cell-adhesion molecule that regulates neural cell growth, migration, and survival during CNS development [80]; however, its role in the normal adult nervous system is not clear. In gliomas, L1CAM is overexpressed and plays a role in tumor invasion [81,82], and is necessary for survival and growth of CD133^+^ cells with stem like properties [83]. Additionally, targeting L1CAM with lentiviral-mediated shRNA interference in CD133^+^ glioma cells inhibited GSC growth and neurosphere formation, and induced GSC apoptosis. L1CAM knockdown decreased OLIG2 expression and upregulated the CDKN1 (also known as p21) tumor suppressor in CD133^+^ glioma cells [83]. ShRNA targeting of L1CAM expression in vivo suppressed tumor growth and increased animals’ survival [83]. L1CAM-mediated signaling conferred radioresistance in GSCs by improving MRE11, RAD50, and NBN (MRN) complex function via the Myc–NBN–ATM axis and by leading to DNA checkpoint activation and DNA repair [84]. Therefore, L1CAM is a promising GSC marker and therapeutic target for GBM.

##### CD44

CD44, a multifunctional Class I transmembrane glycoprotein [85], acts as a specific receptor for hyaluronic acid, promoting migration in normal cells, and is highly expressed in several cancer types [86] and is used to identify CSCs in other tumor types [87,88,89]. Anido and colleagues [57] demonstrated that CD44^high^/ID1^high^ cells were located in the perivascular niches of GBM and possessed stem-cell characteristics. They also showed that TGF-β pathway inhibition decreased the CD44^high^/ID1^high^ population through the repression of ID1 and ID3 levels and prevented tumor initiation [57]. Additionally, high expression of both CD44 and ID1 conferred poor prognosis to GBM patients and were inversely correlated [57]. These results demonstrated that both CD44 and ID1 could be used to identify GSCs.

##### S100A4

Recently, S100A4 was identified as a new biomarker of GSCs [90]. The majority of S100A4^+^ glioma cells were located in perivascular niches and were enriched in cells with characteristics of GSCs. Therefore, S100A4 is a central node in a molecular network that controls stemness and epithelial-to-mesenchymal transition in GBM, suggesting S100A4 as a candidate therapeutic target.

Overall, no individual marker is sufficiently robust to identify GSCs, because of the intra- and intertumor heterogeneity of GSCs. Therefore, using panels of molecular markers and searching for new antigens on the surface of GSCs should improve the purity and specificity of this GBM cell population, and resolve some of the controversies of the current in vitro and in vivo studies.

#### 3.1.2. Side Population

The side-population (SP) assay has been used to identify and isolate CSCs. The SP is a subset of cells with differential efflux activity compared to the main cell population that express high levels of stemness-related genes and are able to generate multiple lineages [35]. Isolation of the SP is based on the capacity that stem cells have of exporting the DNA-binding Hoechst 33,342 dye. This is due to the high expression levels of ATP-binding cassette (ABC) transporters, MDR1 (ABCB1), and BCRP (ABCG2) in stem cells [91,92], which bind ATP and use energy to transport several molecules across the plasma membrane. To identify the SP, cancer cells are stained with Hoechst 33,342 dye, analyzed by flow cytometry, and physically separated from the non-SP by FACS. Two emission wavelengths are used and the small non-stained cell population corresponds to the SP. A criticism of the method is contamination by non-CSCs [93].

#### 3.1.3. Methods of GSC Isolation/Enrichment and Culture In Vitro

There are three most accepted methods of enriching and growing GSC cells: (i) as non-adherent neurosphere cultures [94]; (ii) as an adherent monolayer [46]; or (iii) as organoids [95]. The neurosphere-forming assay is the most widely used. It is similar to those used for culture of NSCs, where cells are cultured in serum-free stem-cell media with specific supplements (commonly, L-glutamine, B27, N2, and the growth factors b-FGF and EGF) [36] (Figure 1). Neurospheres derived from primary tumors express neural precursor markers such as NESTIN, CD133, SOX2, MSI1, and BMI1 [31,96]. Despite the extensive use of the neurosphere-forming assay, this method presents disadvantages. One of them is related to the low efficacy (1 to 30%) with which it can establish GSC lines from primary tumors, because the cells may spontaneously undergo differentiation and/or apoptosis during serial passages [46]. Another limitation is that only a small percentage of cells within a neurosphere are true GSCs, while the majority of cells are partially or fully differentiated progeny [97]. Additionally, it has been shown that the selection of GSCs based on neurosphere culture fails to recapitulate the heterogeneity of the original tumor in vivo, as assessed by gene expression, differentiation capacity, and histological morphology [32,98,99,100].

GSCs grown as monolayers of adherent cells in laminin-coated cell culture plates in serum-free media supplemented with growth factors (Figure 1) can be cultured for at least 1 year (>20 passages) without losing their stem-cell properties and tumor-initiation capacity [46]. The cells in such cultures express NSC markers such as NESTIN, SOX2, and OLIG2. These cells have the ability to differentiate into various lineages, including neuronal and glial, and are highly tumorigenic when implanted into the brains of immunodeficient mice [46]. There is a high percentage of true GSCs in the culture, with significantly fewer differentiated or apoptotic cells. A possible explanation for this optimized result is the fact that all cells have equal access to the components of the medium, a phenomenon that does not occur in tridimensional neurosphere cultures, in which there are gradients of access to factors, and the center of the neurosphere may also become necrotic.

However, both methods fail to represent the tumor architecture and various microenvironments. In this context, 3D GSC organoid culture systems (Figure 1) emerged [95]. This system allows the long-term growth of GSCs from diverse sources (e.g., specimens of human origin, including patient-derived primary cultures, or genetically engineered glioma models), which display regional heterogeneity and recapitulate hypoxic gradients. Moreover, the orthotopic implantation of these organoids resulted in tumors that more closely resembled the original tumor when compared with those from neurosphere cultures from the same patient. More recently, some authors improved this model to better mimic the tumor microenvironment, namely various host-cell interactions. For example, Linkous, et al. [101] proposed the co-culture of GSCs, isolated from patient samples, with human cerebral organoids (the GLICO (glioma cerebral organoids) model). This model carefully mirrors the cellular organization of human brains by culturing human embryonic stem cells (hESCs) or induced pluripotent stem cells (iPSCs) in a way that leads to the formation of a primitive brain. These “mini-brains” display some of the characteristics and important structures of human brains, like a primitive ventricular system, a proliferative zone of NSCs, and a differentiated choroid plexus, and display glial and neuronal components, myelination, and dendrodendritric synapses [101]. The co-culture of GSCs with these fully formed cerebral organoids resulted in stimulation of GSCs which homed into the organoids with deep invasion and proliferation, leading to the creation of tumors that were phenotypically and genetically similar to the original one. Indeed, important genetic features like *EGFR* amplification, which is frequently lost in 2D cultures, are maintained in this model. The authors da Silva, et al. [102] reported a similar model using mouse-derived embryonic stem cells (mESCs) instead of human-derived, which created a primitive neuroepithelial structure to which tumor spheroids almost instantly fused [102]. Following this line of research, other studies have proposed alternative methods that allow the problem to be approached from a different point of view—the oncogenic process from the beginning [103,104]. For this, CRISPR/Cas9 technology is used to express oncogenes and/or block tumor-suppressor genes’ activity within cerebral organoids in a time-controlled manner, which leads to the spontaneous formation of tumors in more complex systems that better mimic true tumors [103,104].

In summary, there are still many challenges that need to be overcome to mimic GBM, namely the interaction of GSCs with normal cells. Some other challenges are (i) current culture conditions enrich the population of GSCs towards EGFR- and FGFR-expressing cells by adding EGF and bFGF supplements, which likely limit the original tumor’s heterogeneity; (ii) the lack of a blood–brain barrier and endothelial cells to mimic the brain vasculature, and absence of immune cells to mimic the tumor–immune cells interaction; and (iii) variability between assays hampers suitable high-throughput capabilities and may thus make them clinically unfeasible. In addition, these methods were optimized for GSC isolation/enrichment, which, while being crucial to the study of GSC-specific phenomena relevant for GBM pathophysiology, also have the disadvantage of not properly reflecting the intrinsic heterogeneity of GBM at cellular, molecular, and metabolic levels. Even the recently developed methods to produce organoids, besides being time-consuming, fail to represent the six characteristic cellular layers present in the cortex, representing only the deeper ones. Moreover, the genetic manipulation of organoids to induce spontaneous tumors might miss some unknown but crucial GBM molecular drivers that thus will reduce their representation. More studies are needed to understand whether organoids are able to support inferences about the tumorigenic capacity of these cells, and to validate the promising results obtained so far. Globally, despite its potential drawbacks, the in vivo limiting dilution assay is still the gold standard experiment for assessing GSC tumorigenicity.

## 4. GSC Molecular Features Amenable for Therapeutic Intervention

Conventional treatments of GBM based on radiotherapy and chemotherapy can lead to a transient elimination or reduction of the tumor bulk. However, almost all GBM tumors recur, possibly due to an increase in the percentage of GSCs [105], as these cells are at the top of the hierarchy that initiates and maintains the tumor even after treatment [106]. In order to effectively eliminate GSCs, it is crucial to understand the molecular and cellular mechanisms underlying their function, such as their signaling pathways and their interactions with the microenvironment.

### 4.1. Major Signaling Pathways in GSCs

**Figure 2:**
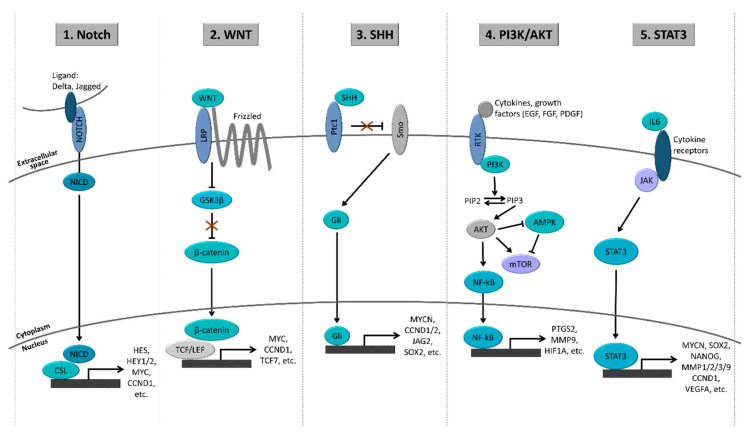
Simplified scheme of critical signaling pathways involved in glioma stem cell (GSC) maintenance. GSCs co-opt several signaling pathways that are also crucial in normal stem cells (e.g., Notch, WNT, SHH, PI3K/AKT, and STAT3 pathways), which hinders a straightforward distinction between cancer and normal stem cells.

In order to maintain an undifferentiated state and increase their survival, GSCs frequently co-opt developmental programs. Some signaling pathways with crucial roles during the normal development have been consistently associated with GSC maintenance, such as the Notch, WNT, SHH, PI3K/AKT, and STAT3 pathways (Figure 2). These pathways may be activated through a combination of genetic and epigenetic alterations, in addition to microenvironmental cues.

#### 4.1.1. Notch Pathway

The Notch family of proteins is part of an evolutionarily well-conserved pathway that is involved in normal development, adult stem-cell maintenance, and tumorigenesis in multiple organs, including the brain [107]. The Notch receptors (NOTCH 1–4), their ligands (JAG1/2 and DLL1/3/4), and the downstream targets HES1 and HES2 are commonly overexpressed in glioma cell lines and primary GBM samples [108]. In astrocytes, the Notch activation stimulates them to acquire a stem like state with increased proliferation [109]. In neural stem-like cells, the knockdown of NOTCH1 by short hairpin RNAs (shRNAs) decreased the expression of NESTIN and CD133 and the formation of neurospheres [109]. In vitro, GBM-derived neurosphere cultures with GSI-18, a Notch inhibitor, showed decreased neurosphere formation and clonogenicity [110,111], reduced expression of *CD133*, *BMI1*, *OLIG2*, and *NESTIN* [110], and increased sensitivity to radiotherapy [112]. Additionally, in vivo delivery of GSI-18 effectively blocked tumor growth, and significantly prolonged animal survival [111]. Furthermore, the inhibition of Notch signaling by GSIs in patient-derived GSCs led to a decreased proliferation and self-renewal ability of these cells, and an increase in their differentiation [113]. It was demonstrated that the use of siRNAs to target *NOTCH1* resulted in decreased proliferation of GSCs in vitro, and a significant delay in the growth of tumors [114]. Different classes of drugs therapeutically targeting Notch, such as γ-secretase inhibitors, receptor/ligand antibodies, and Notch transcription complex inhibitors (e.g., LY3039478/JMSD194, nirogacestat, AL101, demcizumab, enoticumab, brontictuzumab, tarextumab, and CB-103), have been tested in several clinical trials for other (non-glioma) tumors (reviewed in Reference [115]). In GBM, Saito, et al. [116] studied the effects of Notch pathway inhibition in 16 GSC cell lines using various γ-secretase inhibitors (RO4929097, DAPT, and BMS-708163). The results demonstrated that only proneural GSCs were sensitive to γ-secretase inhibitors, and that several components of Notch signaling were highly expressed in proneural GSCs. Additionally, they found that γ-secretase inhibitors impaired GSC maintenance and induced GSC differentiation. Finally, analysis of The Cancer Genome Atlas expression data identified a large percentage (43.9%) of GBM tumors with proneural signatures showing high Notch pathway activation [116]. Another study showed that a combinatory treatment of RO4929097 with standard-of-care TMZ plus radiation therapy decreased 3D spheroid growth, cell proliferation, and expression of stemness markers (*CD133*, SOX2, and NESTIN), leading to a marked reduction in clonogenic survival of primary and established glioma cell lines [117]. Moreover, this combinatory treatment reduced tumor growth and prolonged animals’ survival [117]. Recently, Yu, et al. [118] demonstrated that NOTCH1 is involved in the chemotherapy resistance and tumor recurrence of GSCs [118]. Additionally, it was shown that tenascin-C, an extracellular matrix protein prominent in malignant glioma, might be a potential mechanism of Notch activation in GSCs [119].

Taken together, these studies strongly indicate that Notch pathway activation is essential to maintenance of the GSCs subpopulation, and that this pathway is a potential therapeutic target for blocking GSCs’ growth. In clinical trials, only RO4929097 has been tested for glioma/GBM patients (NCT01122901, NCT01119599, NCT01189240, and NCT01269411 clinical trials). Notch inhibition via the ϒ-secretase inhibitor RO2929097 emerged as a potential therapeutic option based on the modulation of GSCs and a possible anti-angiogenic effect [120]. A Phase I clinical trial for patients with newly diagnosed malignant glioma (anaplastic astrocytoma or GBM) was performed using RO4929097, given together with TMZ and radiation therapy (NCT01119599 [120]). The results demonstrated that the combination of RO4929097, TMZ, and radiation therapy was well tolerated, and no dose-limiting toxicities were observed. Treated tumors presented a significant decrease in the expression of the Notch intracellular domain and cell proliferation. Interestingly, patient-specific organotypic tumor explant cultures revealed a specific decrease in the CD133^+^ GSCs upon a single treatment with TMZ. However, the drug presented variable blood–brain barrier penetration and tumor recurrence occurred, associated with alterations in angiogenesis signaling pathways [120]. Another Phase II trial evaluating RO4929097 as a single agent was also initiated for patients with recurrent or progressive GBM (NCT01122901). Additionally, two other clinical trials were initiated (NCT01189240 and NCT01269411): one Phase I trial studying the side effects and best dose of RO4929097 in treating patients with recurrent invasive gliomas (NCT01269411), and another Phase I/II clinical trial evaluating the side effects and the best dose of RO4929097 when given together with bevacizumab in patients with progressive or recurrent malignant glioma (NCT01189240 [121]). However, production and development of RO4929097 was later interrupted, and these three clinical trials were terminated prematurely without reaching the studies’ endpoints.

#### 4.1.2. WNT Pathway

The WNT signaling pathway is an evolutionarily conserved pathway that plays a crucial role during embryogenesis and adult stem cells by controlling several mechanisms such as cell polarity, cell specification, and tissue homeostasis. In the brain, besides its critical role during its development, this pathway is involved in neurogenesis as well as in controlling the homoeostatic proliferation and self-renewal of NSPCs in the adult subventricular zone [122,123,124]. Taking this into account, it is not surprising that this pathway has been described to be deregulated in cancer, including GBM, and to have an important role in CSC maintenance as well as in resistance to chemotherapy and radiotherapy [125]. Indeed, a transcriptional study reported that although members of the WNT signaling pathway are expressed in both GSCs and NSCs, some are dysregulated in GSCs, leading to WNT overactivation in these cells [126]. Moreover, several members of the WNT pathway have been implicated in GSCs’ self-renewal capacity. We recently showed that WNT6, a ligand of the WNT pathway, increases the self-renewal capacity of GSCs and is associated with stem-cell-related genes and patients’ shorter overall survival [127]. Silencing of WNT6 in GBM cells significantly decreases their capacity to form neurospheres and increases sensitivity to temozolomide (TMZ), leading to decreased tumor aggressiveness in vivo and prolonged overall survival in mice [127]. Interestingly, we demonstrated that in GBM, WNT6 is transcriptionally regulated by HOXA9 [128], a member of a highly conserved family of transcription factors with a crucial role during development and expressed in normal adult stem cells. Hu, et al. [129] reported that WNT5a, another ligand of the WNT pathway, is transcriptionally regulated by PAX6/DLX5, leading to GSCs’ differentiation into endothelial-like cells, ultimately serving as a niche supporting the growth of invasive GBM cells and GSCs self-renewal. Moreover, Binda, et al. [130] showed that WNT5a overexpression is associated with stem-cell characteristics and that its inhibition decreased GSC migration/invasion in vitro and in vivo. WNT3a, also a WNT ligand, is highly expressed in GSCs when compared to their differentiated counterparts [131]. Interestingly, WNT3a silencing in GSCs reduced their clonogenic capacity [131]. Moreover, Kaur, et al. [132] showed that WNT1 and WNT3a silencing in GSCs increased their sensitivity to TMZ and that WNT3a overexpression in a low-grade glioma cell line turned these cells tumorigenic in SCID mice. Frizzled 4, a receptor of the WNT pathway, was also described to control both invasiveness and stemness of GSCs [133]. PLAGL2, an activator of the WNT/β-catenin pathway, promoted self-renewal of GSCs and strongly suppressed their differentiation [134]. In addition, it was recently shown that WNT pathway activation in GSCs is responsible for the dysfunction of MHC Class I and antigen-processing machineries in these cells, reducing the activation of cytotoxic T cells and the elimination of GSCs [135]. Interestingly, Liu, et al. [136] used single-cell RNA-seq to reveal that the WNT pathway is activated and leads to stemness and chemoresistance in circulating tumor cells.

Taking these data into account, therapeutic agents targeting this pathway might effectively deplete GSCs or cause their differentiation and improve the prognoses of GBM patients. Indeed, several inhibitory compounds have shown relevant results in the context of GBM preclinical models. Interestingly, some of these inhibitors are in clinical trials (up to Phase III), but in the context of other tumor types. For example, WNT974 (formerly known as LGK974) has demonstrated promising results in vitro [137,138,139], also being well tolerated by mice and patients [138,139,140]. In fact, in vitro, WNT974 reduced *NANOG* expression in GBM cells and the subpopulation of CD133^+^ GSCs [137]. Moreover, this WNT inhibitor induced glial differentiation and suppressed GBM cells’ clonogenicity. Another study demonstrated that WNT974 restored TMZ sensitivity in in vitro GBM models, contributing to MGMT silencing [138]. Importantly, it was demonstrated that WNT974 is able to cross the blood–brain barrier (BBB) of healthy mice [140], which is of great importance in the context of GBM treatment. Another example is the use of inhibitors of Aurora-A kinase (AURKA), which stabilize the β-catenin destruction complex. For example, both alisertib and TC-A2317 led to the inhibition of the growth and induced the differentiation of GSCs [141]. Moreover, alisertib significantly increased the overall survival of mice implanted with GSC lines [141]. However, while alisertib reached Phase III clinical studies in the context of patients with relapsed or refractory peripheral T-cell lymphoma, it produced no positive effects in the progression-free survival of these patients [142]. Despite these discouraging results, alisertib remains under clinical investigation in other tumor types, both as monotherapy and in combinatorial regimens, showing encouraging results in lower-phase studies [143,144,145]. PRI-724 (ICG-001), a β-catenin/TCF transcription inhibitor, decreased the sphere formation and migration capacity of GSCs [146], and is also under investigation in a Phase II clinical trial for the treatment of patients with other tumor types. Overall, there are enough data supporting the hypothesis that WNT inhibitors, alone or in combination with chemotherapy and/or radiotherapy, might be more effective in the treatment of GBM patients, despite none of the discovered WNT inhibitors having been tested in these patients to date.

#### 4.1.3. Sonic Hedgehog (SHH) Pathway

As with the Notch and the WNT pathways, the SHH pathway is essential for normal brain development and NSC survival, and regulates neural progenitors proliferation and self-renewal in the adult cerebellum [147]. Moreover, the SHH pathway is thought to be activated in primary GBM and glioma cell lines [148], leading to the overexpression of some drug efflux molecules (e.g., ABCB1, ABCG2 [BCRP], and ABCC1 [MRP1]), and MGMT and BMI1 [149,150], which may explain its association with therapy-resistance. GLI1 (glioma-associated transcription factor 1), a target of SHH signaling first identified in human glioma [151], is a modulator of the expression of stemness genes and self-renewal of CD133^+^ GSCs, and plays a key role in tumorigenesis [152]. Activation of SHH signaling was found to be associated with shorter survival of patients whose tumors expressed wild-type *PTEN*, suggesting a relevant association between SHH signaling and molecular profile in GBM [153]. Interestingly, the inhibition of HDAC6 was reported to induce GSC differentiation and apoptosis, and decrease their DNA damage repair capacity via the inactivation of the SHH pathway [154]. Cyclopamine, an inhibitor of the SHH pathway that regulates the active and inactive forms of Smoothened (SMO) protein, reduced the formation of neurospheres and enhanced the response to TMZ-based chemotherapy and radiotherapy [155,156]. Moreover, cyclopamine led to the depletion of CD133^+^ and NESTIN^+^ cells, and the reduction of Hoechst 33,342 SP [155]. In vivo, cyclopamine reduced the tumor volume of intracranial neurospheres xenografts [156]. NVP-LDE-225 (erismodegib or sonidegib), an SMO inhibitor, significantly inhibited cell viability and neurosphere-formation capacity in GBM cells, and the expression of pluripotency-associated proteins (e.g., NANOG, POU5F1, and SOX2) [157]. GDC-0449 (vismodegib), another inhibitor of SMO, was described to significantly reduce the in vitro clonogenicity capacity of GBM cells and the tumorigenic capacity of xenograft GBM models (using both intracranial and subcutaneous models) [158]. Vismodegib is now being tested in a Phase II clinical trial in patients with recurrent GBM after the conventional therapeutic surgery for recurrence removal (NCT00980343). On the other hand, Jin, et al. [159] demonstrated that ID1 promoted the self-renewal of GSCs by simultaneously activating the WNT and SHH pathways, and that WNT and SHH combinatorial inhibition together with BMP treatment significantly suppressed GSCs self-renewal. Similarly, it was demonstrated that the combinatorial treatment of sonidegib and a PI3K inhibitor (NVP-BKM120; buparlisib) significantly decreased the viability of PTEN-deficient GBM neurospheres [160]. Moreover, these authors showed that only this combinatorial regimen was able to significantly reduce the tumor growth in intracranial GBM mouse models [160]. In line with this, it was demonstrated that NVP-BEZ-235 (dactolisib), a dual inhibitor of PI3K and mTOR, cooperates with sonidegib in inhibiting the self-renewal capacity of GSCs, in reducing the expression of pluripotency-maintaining factors (e.g., NANOG, POU5F1, and SOX2), and in inhibiting mouse tumor growth, showing superior effects than either drug alone [161].

Taken together, despite the positive results observed for some of these SHH inhibitors in clinical trials in the context of other tumor types, some patients presented resistance to this strategy (e.g., SHH medulloblastoma patients developed a point mutation in SMO upon treatment, disrupting the ability of sonidegib or vismodegib to bind to SMO [162]). Nonetheless, several SHH inhibitors are already FDA-approved (e.g., vismodegib, sonidegib, and glasdegib) in the context of some SHH-driven tumor types. In GBM and other tumor types, where the SHH pathway presents an important role for GSC maintenance, the targeting of SHH in combination with other drugs might be more suitable, as has been demonstrated in vitro and in vivo [159,160,161]. The great number of ongoing clinical trials testing combinatorial regimens that include SHH inhibitors supports this idea. Indeed, in GBM, glasdegib (PF-04449913), another SMO inhibitor, is being tested in combination with TMZ in Phase Ib/II in newly diagnosed GBM (recruiting status; NCT03466450), with the rationale that the inhibition of the SHH pathway will interfere with GSCs and endothelial migration, as no preclinical data using this drug in GBM models have been published so far.

#### 4.1.4. PI3K/AKT Pathway

The PI3K/AKT signaling is one of the most studied pathways in gliomas, and is often found to be upregulated in GSCs, conferring a high proliferation index and cell survival of this cell subpopulation [163,164]. In GSCs, CD133 phosphorylation at the tyrosine 828 residue in the C-terminal cytoplasmic domain induces the direct interaction between CD133 and p85 (a PI3K regulatory subunit), which leads to the preferential activation of the AKT pathway in these cells [165]. In this way, CD133 silencing inhibited the activity of the PI3K/AKT pathway, reducing GSCs’ self-renewal and in vivo tumorigenicity, which was rescued when CD133 WT was expressed, but not when p85-binding deficient mutant was expressed [165]. These data support the link between PI3K/AKT and GSCs—at least GSCs expressing CD133^+^—and the potential importance of this pathway for the maintenance of the stem phenotype. Malignant gliomas, particularly GBM, frequently display *EGFR* amplification and/or overexpression, providing the rationale for the use of drugs that target this pathway. In vitro studies have shown that the use of EGFR inhibitors (erlotinib, gefitinib, and AG1478) can lead to a decrease in GSC neurosphere formation and proliferation [164,166]. The pharmacological inhibition of AKT also results in the disruption of neurosphere formation [163,167]. Curiously, AMPK, a negative regulator of mTOR (one of the downstream molecules activated by the PI3K/AKT pathway) [168], was recently described as essential for GSC growth in vivo, probably via the regulation of HIF1α and GABPA transcription through CREB1 [169]. Rapamycin (an mTOR inhibitor) treatment promoted GSC differentiation and increased in vitro radiosensitivity [170]. Moreover, a novel dual PI3K/mTOR inhibitor, NVP-BEZ235, was also shown to increase the radiosensitivity of GSCs in vitro, in association with a synergistic increase of cell-cycle arrest and apoptosis, and a decrease of the DNA repair capacity of these cells [171]. Unfortunately, the use of these drugs in clinical trials for GBM has not been particularly successful. These disappointing results can be partly explained by the redundancy of upstream signaling mechanisms that result in PI3K/Akt activation (e.g., EGFR, c-MET, and PDGFR), as well as by some intracellular mediators acting downstream of PI3K/AKT that prevent successful cytotoxic activity, or simply due to tumor heterogeneity. To add more complexity to the matter, microenvironmental factors can significantly modulate the activity of many of these drugs.

#### 4.1.5. STAT3 Pathway

The signal transducer and activator of transcription 3 (STAT3) is an important transcriptional regulator involved in CNS development, stem-cell maintenance, proliferation, and tumorigenesis [172]. STAT3 inhibition with specific inhibitors (e.g., S3I-201 and STA-21) or short hairpin RNA (shRNA) led to a decrease in proliferation of GSCs and neurosphere formation [172]. Upstream of STAT3, interleukin-6 (IL6), erythropoietin (EPO), and Notch signaling (see above) are also found frequently to be activated in the subpopulation of GSCs. The specific inhibition of EPO receptor or IL6 with shRNAs resulted in the inhibition of STAT3, and decreased the cell growth and self-renewal capacity of GSCs [173]. Additionally, treatment with IL6 antibody decreased the growth of GSC-derived tumors. WP1193, a small molecule and potent inhibitor of the JAK2/STAT3 pathway, was shown to inhibit GSC neurosphere formation [174]. Interestingly, under hypoxic conditions, the transcription factor hypoxia inducible factor 1α (HIF1α) activates the JAK1/2-STAT3 axis and enhances GSC self-renewal [175]. Another potential mechanism of STAT3 activation in GSCs is TRIM8, through PIAS3 suppression [176]. TRIM8 silencing reduced GSCs self-renewal and promoted glial differentiation. Interestingly, the authors also found that STAT3 activation upregulated TRIM8, suggesting a feedback loop. Recently, STAT3, together with integrin-α6, and TET3 were shown to regulate the DNA cytosine 5′-hydroxymethylation of genes important for GSCs, promoting their tumorigenicity and therapy-resistance [177]. Remarkably, and supporting the important role of the tumor microenvironment, nontumorigenic glioma-associated mesenchymal stem cells (MSCs) are able to increase the proliferation and self-renewal of GSCs by secreting IL6 in a paracrine fashion by inducing the STAT3 pathway in GSCs [178]. Moreover, genomic sequencing demonstrated that most of the MSCs (60%) were normal cells recruited to the tumor, 10% might be the result of GSC differentiation, and the remainder presented a mixed genotype. In vitro treatment with napabucasin, a novel small-molecule inhibitor of STAT3, decreased the expression of stemness-associated genes and suppressed GSC neurosphere formation [179]. In vivo, this new STAT3 inhibitor impaired glioma growth [179]. The use of MDC-1112, which was demonstrated to inhibit STAT3 phosphorylation at the serine 727, significantly inhibited the growth of GBM cell lines and their clonogenicity in vitro [180]. In vivo, it was associated with decreased tumor volume and increased mouse survival [180]. WP1066, which is now in a Phase I clinical trial recruiting patients with recurrent malignant gliomas (NCT01904123), showed promising preclinical results [181]. Indeed, a first report demonstrated that WP1066 was able to significantly decrease the viability of GBM cell lines [181]. Mechanistically, the authors demonstrated that WP1066 inhibits STAT3 phosphorylation at the tyrosine 705, inhibiting its nuclear translocation [181]. In a subcutaneous GBM mouse model, WP1066 significantly inhibited tumor growth [181]. Others demonstrated that WP1066 led to an increased survival time of spontaneous glioma mouse models [182], and to the increased radiosensitivity of GSCs implanted in mouse brains [183]. Importantly, WP1066 inhibited GSC cell growth and prolonged the survival of an orthotopic mouse model induced by GSC implantation [184]. STX-0119, another STAT3 inhibitor, demonstrated a stronger in vitro growth inhibitory effect than WP1066 in GSCs [185]. Moreover, the use of STX-0119 significantly reduced the expression of GSC-associated genes (i.e., *CD44*, *NANOG*, *NES*, *CD133*) [185]. Mice orthotopically implanted with GSCs also demonstrated longer overall survival when treated with STX-0119 [185]. Overall, a great number of STAT3 inhibitors have been developed, including peptides, drugs, natural inhibitors, antisense oligonucleotides, and decoys, but only a few have been tested in GBM models, and only a small portion tested in GSCs. Other STAT3 inhibitors have the potential to help target this subpopulation of cells, which is highly resistant to conventional therapies. Additional first-in-human studies are necessary to clarify their clinical efficacy.

In summary, given that many of these signaling pathways are also essential for embryonic development and adult stem-cell maintenance, care should be taken when evaluating inhibitory drugs targeting these pathways. Although most of the drugs that reached the first phases of clinical trials were shown to be well-tolerated in adults, their use in younger patients must be well weighted. Bone and hematological toxicities have been observed in some cases, emphasizing the need for a rigorous cost–benefit assessment before entering more advanced phases. The highly described crosstalk and compensation mechanisms observed between the above-mentioned pathways are a possible reason for unsuccessful or incomplete responses in the clinical trials. Notwithstanding, some of these drugs have already been approved in the context of other tumor types. Thus, one might hypothesize that these drugs may not efficiently cross the BBB and would thus require higher dosages to be effective, which might be associated with increased systemic toxicities, or may need to be combined with more advanced drug-delivery systems. On the other hand, some of these drugs were initially tested in other tumor types, in some cases presenting excellent results and reaching FDA approval, and are only now entering the first phases of clinical trials with GBM patients. Moreover, targeting only GSCs, although critical, is likely to be insufficient to achieve complete and clinically meaningful responses, as (i) differentiated GBM cells may convert themselves into GSCs (detailed in Section 1 and Section 3); (ii) particular tumor microenvironments promote the self-renewal of GSCs and non-GSCs (detailed in Section 4.2 below); and (iii) GBM are highly heterogeneous, and GSCs also contribute to this characteristic (detailed in Section 3). In this context, combinatorial regimens that target both GSCs and non-GSCs are preferable to ideally ensure a more effective and possibly complete elimination of the tumor. In addition, it might be interesting to develop agents with the capacity to simultaneously target multiple pathways, or to identify a safe combination of drugs already available that may contribute to the elimination of these distinct subpopulations of GSCs, which might be further combined with conventional therapies to target differentiated cells.

### 4.2. Targeting GSCs Vascular and Hypoxic Microenvironment

In order to better understand how the different signaling pathways mentioned above regulate GSC biology, it is crucial to study the various niches in which these cells reside, as increasing evidence suggests that the tumor microenvironment is responsible for at least part of the variability observed in GSC clones [186]. The majority of GSCs are located in vascular niches and other tumor microenvironments that regulate nutrient and oxygen supply [187], where they maintain a close contact with CD34^+^ endothelial cells [187,188,189]. These endothelial cells play an important role in the pathophysiology of GBM and provide essential signals for NSCs’ self-renewal [190]. GSCs present in the tumor interact directly or indirectly with the tumor vasculature and with endothelial cells, inducing self-renewal and proliferation of GSCs both in vitro and in vivo [188]. It is believed that bFGF, TGF-β, and nitric oxide molecules and SHH and Notch pathway factors produced by endothelial cells lead to the activation of GSCs [57,189,191,192,193,194]. Furthermore, Lathia, et al. [195] described that laminin α2, a protein from the extracellular matrix enriched in the tumor vasculature, is also able to induce GSC self-renewal. In addition, GSCs are also able to interfere with the tumor vasculature in an intricate bidirectional crosstalk by promoting angiogenesis through the release of high levels of VEGF and other angiogenic factors that promote the migration of newly endothelial cells into the tumor [196,197]. Additionally, tenascin-C modulates the GSCs’ secretome, leading to an increased expression of a range of angiogenic factors [198]. Moreover, and even more surprising, GSCs are able to generate the majority of pericytes and endothelial cells present in the tumor, suggesting that these cells have an important role in maintaining their own tumor microenvironments and niches [199,200,201,202,203]. Additional studies have also demonstrated the endothelial progenitor-like characteristics of CD133^+^ GSCs [204,205,206]. This phenomenon of vascular mimicry is considered a new model of tumor microcirculation distinct from classical tumor angiogenesis, since it does not depend on endothelial cells to supply sufficient blood for tumor growth [207], and has been associated with high tumor grade, tumor progression, invasion, and poor prognosis in patients with malignant tumors, including GBM [208]. It has been demonstrated that both Nestin and CD133 are biomarkers common to GSC and vascular mimicry-initiating stem cells [96,209]. Finding new ways to therapeutically target this vascular process may also prove useful for patients.

It is also well-recognized that hypoxic tumor niches are critical hubs of several processes relevant for GBM pathophysiology. For example, hypoxia can promote angiogenesis through the expression of hypoxia-inducible factors (HIFs) [210]. Li, Bao, Wu, Wang, Eyler, Sathornsumetee, Shi, Cao, Lathia, McLendon, Hjelmeland and Rich [39] demonstrated that HIF2α and multiple HIF-regulated genes are preferentially expressed by GSCs when compared to non-GSC tumor cells and NSPCs. These authors also showed that HIF2α co-localizes with GSC markers in patients’ tumor samples. In vitro, they demonstrated that HIF targeting resulted in GSCs’ self-renewal, proliferation, and survival inhibition. In vivo, the targeting of HIFs decreased the tumor-initiation capability of the GSCs. Additional studies further demonstrated the importance of hypoxia by showing that it is able to promote the self-renewal capability of both GSCs and non-GSCs [211]. Moreover, HIF2α increased the tumorigenic potential of non-GSCs [211]. Overall, these studies demonstrated that hypoxic regions increase the GSC subpopulation, promoting the acquisition of a stem-like state. Another study described the increased expression of proinflammatory proteins in hypoxic regions from human GBM tumor samples, when compared to peritumoral and normal samples [212]. Indeed, GSCs cultivated in hypoxic conditions presented increased levels of HIF1α and proinflammatory molecules (e.g., NF-kB, P2RX7, and CXCR4) and increased invasion and migration capabilities [212].

Since the microenvironment is able to promote GSC maintenance, and, as discussed, there is a constant bidirectional crosstalk between tumor cells and their microenvironments, this interaction should be further studied and explored from a therapeutic perspective. Indeed, Zhou, et al. [213] demonstrated that the targeting of GSC-derived pericytes is able to disrupt the blood–tumor barrier, and leave the blood–brain barrier intact, enhancing drug effusion around tumor cells. Globally, these studies highlight the existence and relevance of a complex crosstalk between GSCs and the various different niches in the tumor microenvironment in which they reside, dynamically changing during the various phases of GBM initiation, progression, and recurrence. Further knowledge on this crosstalk will allow more efficient targeting with novel therapies.

### 4.3. Targeting GSCs by Inducing Differentiation

In addition to more efficiently eradicating GSCs, some groups have been exploiting the possibility of promoting differentiation of GSCs as a promising and less toxic therapeutic strategy. For example, bone morphogenetic proteins (BMPs) are members of the TGF-β family of secreted ligands that appeared as potential soluble factors for glioma treatment. In very early stages of embryonic CNS development, BMP2/4 are responsible for neuroepithelial proliferation, but in later stages of development they induce neuronal and astrocytic differentiation of NSCs [214,215,216,217]. In GBM, BMPs function as a differentiation signal [218]. For this reason, BMPs have been used as pro-differentiating factors for GBM treatment, being able to reduce GBM cell growth and promote astroglial differentiation [219,220]. It was also demonstrated that glioma cells exposed to BMP4 showed a significant reduction in the proportion of GSCs, and the in vivo delivery of BMP4 effectively blocked tumor growth [219]. Another study demonstrated that BMP7 is able to inhibit GBM growth in vitro and in vivo [221], and to block GSC self-renewal, proliferation, and tumor initiation [222]. It is noteworthy that 20% of GBM tumors present epigenetic silencing of BMPR1B (BMP receptor) due to promoter CpG methylation [223]. Therefore, a subset of GSCs is able to escape from the differentiation induced by BMPs. Together, these results suggest that BMPs or other differentiation-modulating agents could be used as a potential therapeutic approach for brain cancer. Indeed, metformin (an antidiabetic agent) was identified as a FOXO3 activator [224], and the activation of FOXO3 induced GSC differentiation and reduced tumorigenicity. In vivo, it was demonstrated that glioma-bearing mice treated with metformin presented tumor-formation inhibition, depletion of GSC subpopulation, and prolonged median survival [224]. Future studies exploring new and clinically relevant targets that can be used to target GSC differentiation, and understanding their underlying mechanisms, may prove critical to improving GBM treatment.

## 5. Conclusions and Future Perspectives

The discovery of GSCs in the past two decades has been an exciting breakthrough and has helped to elucidate important aspects of brain tumor biology, contributing to the understanding of the concept of glioma heterogeneity and mechanisms of therapy resistance. Despite these advances, there are also several challenges ahead, including (i) the lack of a universal marker to identify GSCs; (ii) the fact that the common molecular signaling pathways are shared by GSCs and normal NSPCs; and (iii) the incomplete knowledge about GSCs’ biology and how these cells interact with the tumor microenvironment. Therefore, it is of critical importance to join efforts to improve the isolation and culture methodologies to obtain a purer population of GSCs or, at least, to improve the yields of these approaches. The selection of the GSCs using a combination of several molecular markers is particularly useful, although they do not enrich for a population of GSCs in every GBM. The levels of these markers vary greatly between different brain tumor specimens, since intratumoral and interpatient variations occur. Thus, the implementation of new methods for GSC culture, and the discovery of specific markers and dysregulated pathways in GSCs, will help in producing new therapeutic strategies that specifically target these cells, and ultimately, guide the selection of the appropriate treatment for patients bearing such aggressive tumors. Emergent technologies like single-cell sequencing and functional analysis might be useful to study and better understand not only the tumor heterogeneity, but also the now-well-recognized heterogeneity within GSCs. The CSC theory adds an additional level of complexity that contributes to the malignancy of GBM, in which GSCs should not be considered as an isolated component. Instead, it is important to take into account the fact that GSCs reside in multiple niches influenced by different molecular programs and that the tumor microenvironment has an impact on the maintenance and tumorigenic potential of these cells. Thus, to eliminate GSCs, it will likely be necessary to develop multitargeted approaches.

Recently, immunotherapy has emerged as a potential therapeutic option for cancer [225]. One option is the use of immune checkpoint inhibitors (ICIs), to which, despite showing good results in some cancer types [226,227], most patients still do not respond, likely due to tumor-intrinsic mechanisms of resistance [228]. Interestingly, potential mechanisms may be related to aberrant WNT, Notch, SHH, or STAT3 pathways, which have important roles in GSC maintenance [229,230,231]. This raises the question of whether GSCs, through the upregulation of these developmental pathways, contribute to immune evasion. For now, most of the experiments studying GSCs present a major caveat: they use immunocompromised mice to study the impact of GSCs in tumor initiation, progression, and therapy response. Indeed, only a few recent studies have revealed that GSCs may be part of the mechanism behind the immune-evasion characteristic of GBMs. For example, it was demonstrated that extracellular vesicles released by GSCs block T-cell activation and proliferation in response to T-cell receptor stimulation [232], which might be due do their action on monocyte maturation [233]. Moreover, GSCs secrete periostin (POSTN), which recruits M2-like tumor-associated macrophages (TAMs) and promotes GBM aggressiveness [234]. Others reported that GSCs express multiple immunomodulatory cell-surface molecules [235], including GM-CSF, that induce TAMs development by promoting the survival and differentiation of bone-marrow-derived monocytes [236]. Furthermore, GSCs are reported to downregulate the expression of histocompatibility complex Class I (MHC-I) molecules and antigen-processing machinery (APM) components when compared to non-GSCs, by overactivating the WNT pathway [135]. Others showed that GSCs downregulated the expression of TLR4, and that TLR4 overexpression in GSCs inhibited their proliferation and maintenance [237]. In addition, macrophages accumulating in the tumor’s core and borders induce stemness and chemoradioresistance of GBM cells, which might be due to HB-EGF, IL1β, and pleiotrophin (PTN) secretion [238,239]. Interestingly, PTN silencing in M2-like macrophages decreased their pro-tumorigenic activity when these cells were co-implanted with GSCs [239]. Others demonstrated that the immunosuppressive myeloid-derived suppressor cells (MDSCs) are present in close proximity to GSCs in the tumors of GBM patients [240]. Indeed, co-culture experiments of patient-derived GSCs with MDSCs, when compared to differentiated tumor cells, revealed that GSCs secrete high levels of multiple factors (e.g., macrophage migration inhibitory factor—MIF), which induce MDSC-mediated immune suppression [240]. In the context of the currently scarce existing literature, it seems that GSCs may play a crucial role in the regulation of immune cells through distinct mechanisms, ranging from protein secretion to ligand downregulation, or by activating pro-tumoral immune-related pathways. Future studies are thus needed to better understand this novel layer of complexity regarding the communication between GSCs and immune cells, and how this may impact disease outcome.

In summary, it is clear that (i) GSCs are a subpopulation of GBM cells with an important role for therapy resistance and tumorigenicity; (ii) GSCs hijack developmental pathways to maintain their self-renewal capacity; (iii) GSCs maintain and represent the heterogeneity existing in the tumor; and (iv) GSCs are able to create their own niche by recruiting and/or producing both endothelial and protumoral immune cells, from which they take advantage in return in a still incompletely known positive feedback loop. These findings highlight GSCs as an important therapeutic target for GBM, warranting additional efforts to understand their interactions with other cells of the tumor microenvironment, in order to be able to propose new, effective therapies.
